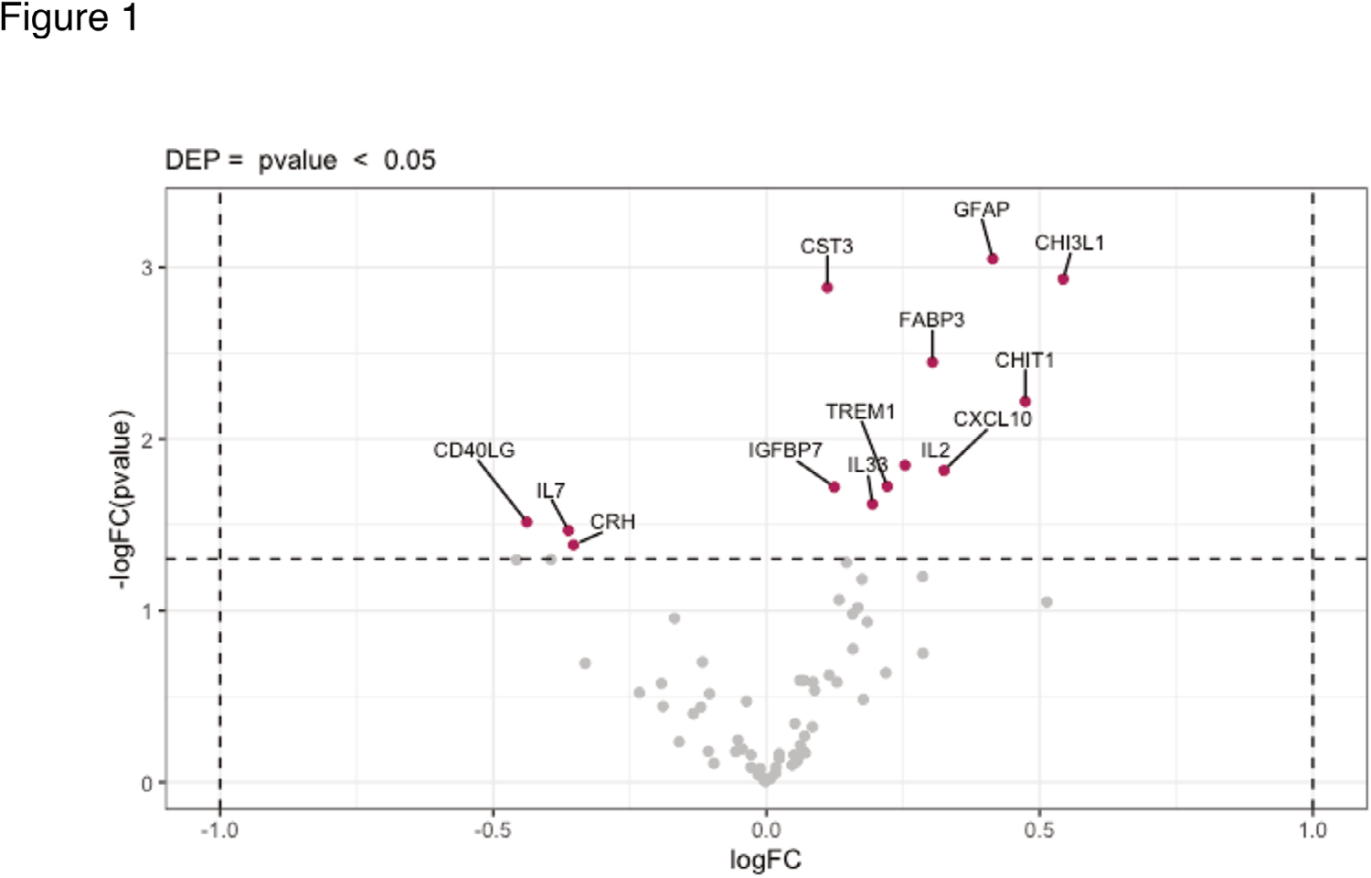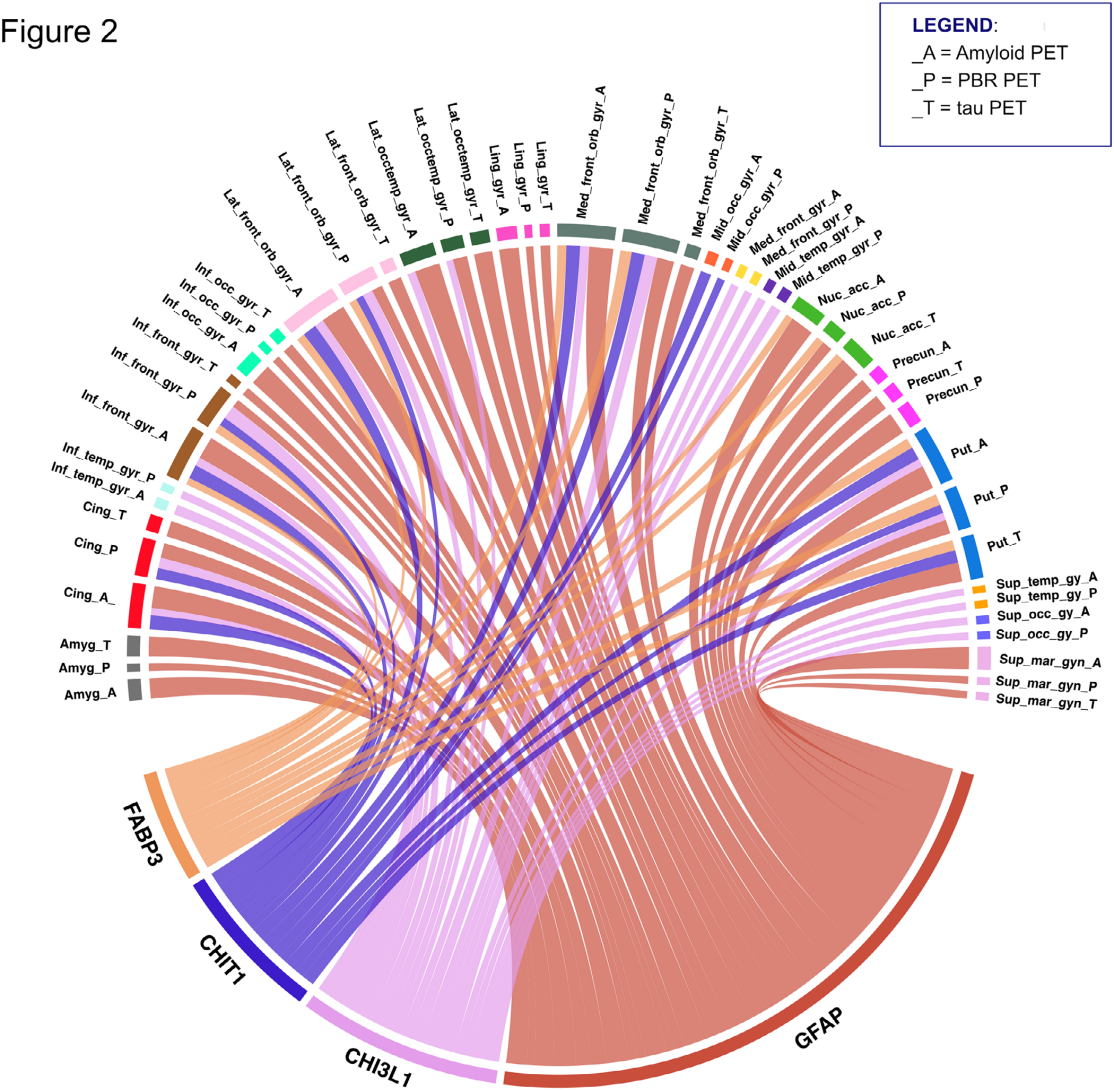# Inflammation links in Alzheimer’s Disease: connecting fluid proteomics and TSPO PET

**DOI:** 10.1002/alz.092999

**Published:** 2025-01-09

**Authors:** Ilaria Pola, Nicholas J. Ashton, Marco Antônio de Bastiani, Wagner Scheeren Brum, Nesrine Rahmouni, Stijn Servaes, Jenna Stevenson, Cécile Tissot, Joseph Therriault, Tharick A. Pascoal, Kaj Blennow, Eduardo R. Zimmer, Henrik Zetterberg, Pedro Rosa‐Neto, Andrea L. Benedet

**Affiliations:** ^1^ Department of Psychiatry and Neurochemistry, Institute of Neuroscience and Physiology, The Sahlgrenska Academy, University of Gothenburg, Mölndal Sweden; ^2^ Department of Psychiatry and Neurochemistry, Institute of Neuroscience and Physiology, The Sahlgrenska Academy, University of Gothenburg, Mölndal, Gothenburg Sweden; ^3^ King’s College London, Institute of Psychiatry, Psychology & Neuroscience, Maurice Wohl Clinical Neuroscience Institute, London UK; ^4^ Wallenberg Centre for Molecular and Translational Medicine, University of Gothenburg, Gothenburg Sweden; ^5^ Federal University of Rio Grande do Sul, Porto Alegre, Rio Grande do Sul Brazil; ^6^ Graduate Program in Biological Sciences: Biochemistry, Universidade Federal do Rio Grande do Sul (UFRGS), Porto Alegre Brazil; ^7^ The McGill University Research Centre for Studies in Aging, Montreal, QC Canada; ^8^ Translational Neuroimaging Laboratory, The McGill University Research Centre for Studies in Aging, Montréal, QC Canada; ^9^ McGill Centre for Studies in Aging, Department of Neurology and Neurosurgery, McGill University, Montreal, QC Canada; ^10^ Lawrence Berkeley National Laboratory, Berkeley, CA USA; ^11^ McGill University Research Centre for Studies in Aging, Montreal, QC Canada; ^12^ Departments of Psychiatry and Neurology, University of Pittsburgh School of Medicine, Pittsburgh, PA USA; ^13^ Institute of Neuroscience and Physiology, Sahlgrenska Academy at the University of Gothenburg, Göteborg Sweden; ^14^ Brain Institute of Rio Grande do Sul ‐ Pontifícia Universidade Católica do Rio Grande do Sul, Porto Alegre, Rio Grande do Sul Brazil; ^15^ Wisconsin Alzheimer’s Disease Research Center, University of Wisconsin School of Medicine and Public Health, Madison, WI USA; ^16^ Dementia Research Centre, Department of Neurodegenerative Disease, UCL Queen Square Institute of Neurology, University College London, London, United Kingdom, London UK; ^17^ Hong Kong Center for Neurodegenerative Diseases, Clear Water Bay Hong Kong; ^18^ Translational Neuroimaging Laboratory, Montreal, QC Canada; ^19^ Montreal Neurological Institute, Montréal, QC Canada

## Abstract

**Background:**

Emerging evidence underscores the importance of neuroinflammation in the progression of Alzheimer’s disease (AD) pathophysiology. Recent studies indicate the involvement of the inflammatory mechanisms both in amyloid‐ β (Aβ) and tau deposition in the brain. Nevertheless, due to the complexity of the immune responses and the intricate interplay between the peripheral and the central nervous systems, identifying biomarkers that reflect the brain´s inflammatory state in AD has been a challenge. For this reason, the characterization of immune‐related proteins in cerebrospinal fluid (CSF) and plasma would contribute to the understanding of the role of neuroinflammation in the progression of AD.

**Method:**

Participants from the Translational Biomarker for Aging and Dementia Cohort (TRIAD) incorporating within the AD spectrum, and with available amyloid Aβ ([^18^F]AZD4694), tau ([^18^F]MK6240) and TSPO ([^11^C]PBR28) PET data (positivity = 2.5 SD > mean ROI‐SUVR of young participants), had plasma (n= 151) samples analyzed with the NULISA technology (Alamar Biosciences^®^). Inflammation‐related proteins (n=72) were selected and included in our analysis, which differential expression was evaluated with linear models (LIMMA) contrasting TSPO groups. After FDR correction for multiple comparisons, the differentially expressed proteins were selected for further analysis, where protein levels were correlated with the PET uptake of 45 anatomical brain regions.

**Result:**

The differential expression analysis unveiled 5 proteins that are in higher concentrations in the plasma of TSPO positive in comparison with TSPO negative participants (Figure 1): GFAP, CHI3L1, CST3, FABP3, and CHIT1. The correlation analysis between the plasmatic proteins and PET uptake showed significant overlapping correlations with TSPO, Aβ and tau PET in brain regions such as inferior frontal gyrus, inferior occipital gyrus and amygdala (Figure 2).

**Conclusion:**

These preliminary findings underscore the relevance of GFAP, CHI3L1, CST3, FABP3, and CHIT1 at proxying immune‐related processes in AD, by linking peripherally quantified proteins to brain pathology, as suggested by the colocalized correlations with amyloid, tau and TSPO PET uptake. Further replication of this analysis on the CSF proteomic data of these participants will support the potential significance of these proteins as valuable indicators of neuroinflammation in AD pathology.